# Clinical characteristics and risk factors of severe myelosuppression in rheumatoid arthritis patients with csDMARD non-adherence: a case series

**DOI:** 10.3389/fphar.2026.1722407

**Published:** 2026-02-26

**Authors:** Xiaoli Pan, Jingqiao Tian, Juan Chen, Yulei Ao, Mei Tian, Anmao Li

**Affiliations:** Department of Rheumatology and Immunology, The Affiliated Hospital of Zunyi Medical University, Zunyi, China

**Keywords:** adverse drug reaction, bone marrow suppression, csDMARDs, medication adherence, methotrexate, rheumatoid arthritis

## Abstract

This study investigated the clinical characteristics and risk factors of bone marrow suppression in rheumatoid arthritis (RA) patients treated with conventional synthetic disease-modifying antirheumatic drugs (csDMARDs) to provide evidence for improving medication safety. We retrospectively analyzed clinical data from 30 RA inpatients with csDMARDs-induced bone marrow suppression hospitalized at the Affiliated Hospital of Zunyi Medical University between August 2022 and January 2025. Methotrexate was part of the treatment regimen for twenty-seven patients, accounting for 90% of the cohort. Medication non-adherence was identified in fifteen patients, representing 50% of cases, with unauthorized dose escalation being the primary pattern. All patients developed severe Grade III to IV bone marrow suppression. Pancytopenia was observed in twenty patients, constituting 66.7% of the total. Common complications included febrile neutropenia, oral mucositis, and gastrointestinal bleeding. Following comprehensive treatment, all patients achieved hematologic recovery and were discharged. Our findings indicate that medication non-adherence, particularly self-driven escalation of methotrexate dosage, is a critical risk factor for life-threatening bone marrow suppression in rheumatoid arthritis patients. These results underscore the necessity of enhanced patient education, strict adherence monitoring, and rigorous hematologic surveillance for high-risk individuals.

## 1 Introduction

Rheumatoid arthritis (RA) is a systemic autoimmune disease characterized primarily by erosive synovitis, which can occur at any age (Rheumatoid arthritis, 2018). As of 2021, the global prevalence of RA was estimated at approximately 17.9 million cases. Epidemiological data indicate that between 1990 and 2021, the global incidence rate increased by 13.2%, with a trend toward earlier onset and expanding affected populations (Jin et al., 2025). In China, the prevalence is approximately 0.42%, with a total of about 5 million patients. The disease is more common in women than in men, with a median age at onset of approximately 52 years. A considerable proportion of patients exhibit moderate to high disease activity (Jin et al., 2017; Yu et al., 2018). Although the exact pathogenesis of RA remains incompletely understood, it is widely believed to involve aberrant immune activation, ultimately leading to progressive joint destruction, deformity, and loss of function. Currently, conventional synthetic disease-modifying antirheumatic drugs (csDMARDs) are consistently recommended as first-line therapy for RA in both domestic and international guidelines (Smolen et al., 2023; Tian et al., 2025). These mainly include methotrexate (MTX), leflunomide (LEF), sulfasalazine (SSZ), hydroxychloroquine (HCQ), iguratimod (IGU). Among these, MTX is the most commonly used csDMARD, frequently administered as monotherapy or in combination with other csDMARDs (Smolen et al., 2023; Tian et al., 2025). However, these agents are associated with various potential adverse effects during treatment, among which bone marrow suppression is one of the common and serious complications (Lien and Tsai, 2022). The occurrence of bone marrow suppression not only significantly increases the economic burden on patients but may also be life-threatening. Therefore, in-depth analysis of the incidence of bone marrow suppression following csDMARD therapy in RA is of great importance. To this end, this study retrospectively analyzed the clinical characteristics of 30 RA patients who developed bone marrow suppression after csDMARD administration, aiming to provide clinical insights for the prevention and management of this serious adverse drug reaction.

## 2 Methods

### 2.1 Patients

A total of 30 patients admitted to the Department of Rheumatology and Immunology at the Affiliated Hospital of Zunyi Medical University from August 2022 to January 2025 were enrolled in this study. The inclusion criteria were as follows: (1) diagnosis of RA based on clinical manifestations and laboratory tests, fulfilling the 2010 ACR/EULAR classification criteria (Aletaha et al., 2010); (2) age ≥18 years, with capacity to provide informed consent; (3) availability of complete and valid clinical data. The exclusion criteria included: (1) comorbid other autoimmune diseases or hematological disorders; (2) pre-existing bone marrow suppression prior to treatment; (3) incomplete clinical documentation.

### 2.2 Data collection

The collected data included patient age, gender, blood type, educational level, place of residence, details of csDMARDs regimens, potential causes inducing bone marrow suppression, comorbidities, laboratory findings, treatment strategies, and clinical outcomes.

### 2.3 Observation indicators

Fasting venous blood samples were collected from participants in the morning. Laboratory testing was performed using a Beckman Coulter AU5800 fully automated biochemical analyzer. The primary laboratory parameters included: Red blood cell (RBC) count (reference range: male 4.3–5.8 × 10^12^/L, female 3.5–5.0 × 10^12^/L); white blood cell (WBC) count (reference range: 4–10 × 10^9^/L); platelet (PLT) count (reference range: 100–300 × 10^9^/L); hemoglobin (HB) level (reference range: male 130–175 g/L, female 115–150 g/L).

### 2.4 Definition of malnutrition

Malnutrition was defined in this study based on a composite of objective clinical and biochemical parameters commonly used in hospitalized patients. A patient was classified as having malnutrition if they met at least one of the following criteria: (1) body mass index (BMI) <18.5 kg/m^2^; (2) serum albumin level <35 g/L; or (3) documented unintentional weight loss >5% of body weight within the preceding 3 months.

### 2.5 Definition of alanine aminotransferase elevation and renal impairment

Alanine aminotransferase (ALT) elevation defined as a serum alanine aminotransferase level exceeding 40 U/L. Renal impairment was defined as a serum creatinine level above the sex-specific upper limit of normal, which is 106 μmol/L for males and 97 μmol/L for females. These diagnostic thresholds were established based on the laboratory reference standards historically employed at our hospital.

### 2.6 Definition of clinical outcomes

For the assessment of treatment response, operational definitions were applied to the terms “Improved” and “Normalized.” Regarding hematological recovery, “Normalized” was defined as all measured parameters (RBC, WBC, PLT counts, and Hb level) returning to within the laboratory’s normal reference ranges specified in the Observation Indicators section. “Improved” was defined as a clear increase from admission baseline in any or all of these hematological parameters after treatment, without fulfilling all criteria for “Normalized.” For hepatic and renal function, “Improved” indicated a notable decrease in serum creatinine (Scr) or alanine aminotransferase (ALT) levels from the admission baseline, while “Normalized” required these values to fall within the normal reference ranges.

### 2.7 Management of adverse reactions

(1) Bone marrow suppression: patients with agranulocytosis were placed on an aseptic diet and administered recombinant human granulocyte colony-stimulating factor (rhG-CSF) to promote granulocyte recovery. Recombinant human interleukin-11 (rhIL-11) was used to stimulate megakaryocyte proliferation and platelet production. Platelet transfusion was administered when necessary. Patients with severe anemia received red blood cell (RBC) transfusion to correct anemia and improve oxygen-carrying capacity. (2) Oral mucositis and ulceration: patients were advised to maintain oral hygiene. Topical treatments were selected based on the severity of lesions, including sodium bicarbonate solution, Kangfuxin liquid, or compound mouthwash containing lidocaine and dexamethasone. (3) Drug eruption: Levocetirizine was administered to alleviate allergic symptoms, and dexamethasone was used to mitigate the inflammatory response. (4) Acute gastric mucosal injury: this condition was managed with acid suppression therapy using proton pump inhibitors (PPIs) in combination with gastric mucosal protectants. (5) Liver injury: reduced glutathione was administered as hepatoprotective therapy. (6) Infection: empiric broad-spectrum, potent antibacterial therapy (e.g., meropenem) was initiated to cover pathogens potentially responsible for life-threatening infections and serious complications. Vigilance for secondary infections was maintained, and the regimen was adjusted accordingly based on etiological findings. (7) Immunomodulatory therapy: intravenous immunoglobulin (IVIG) was administered to elderly patients with severe concurrent infections for anti-infective purposes and to enhance immune function.

### 2.8 Grading of bone marrow suppression

The severity of bone marrow suppression was graded according to the 《Expert Consensus on Outpatient Management of Treatment-Related Bone Marrow Suppression (2025 Edition)》 (Committee, 2025). Bone marrow suppression was classified into Grades I to IV based on the extent of reduction in peripheral blood cell counts:Grade I (mild): Hb below the lower limit of normal to 100 g/L, absolute neutrophil count (ANC) below the lower limit of normal to 1.5 × 10^9^/L, or PLT below the lower limit of normal to 75 × 10^9^/L; Grade II (moderate): Hb <100 to 80 g/L, ANC <1.5 to 1.0 × 10^9^/L, or PLT <75 to 50 × 10^9^/L; Grade III (severe): Hb <80 to 65 g/L, ANC <1.0 to 0.5 × 10^9^/L, or PLT <50 to 25 × 10^9^/L; Grade IV (life-threatening): Hb <65 g/L, ANC <0.5 × 10^9^/L, or PLT <25 × 10^9^/L.

### 2.9 Statistical analysis and ethical issues

Statistical analysis was performed using SPSS 29.0 software. The associations between all baseline characteristics and medication adherence were analyzed using univariate methods. Specifically, independent samples t-test was used for group comparisons of continuous variables with normal distribution (e.g., age), while the Mann-Whitney U test was applied for non-normally distributed data. Group comparisons for categorical variables (e.g., disease duration grouping, educational level grouping) were performed using the chi-square test or Fisher’s exact test, as appropriate. A *p*-value of less than 0.05 was considered to indicate a statistically significant difference. This study was approved by the Ethics Committee of the Affiliated Hospital of Zunyi Medical University.

### 2.10 Consideration of potential confounding factors

While this study highlights medication non-adherence as a key risk factor for severe bone marrow suppression, the potential influence of other clinical confounders must be acknowledged. Impaired renal function can reduce methotrexate clearance, leading to drug accumulation and enhanced toxicity, independent of adherence behavior. Malnutrition may compromise bone marrow reserve and patient resilience to drug effects. Furthermore, pharmacokinetic or pharmacodynamic interactions between csDMARDs (e.g., concurrent NSAIDs potentially reducing MTX renal excretion) or with other concomitant medications could synergistically increase myelotoxic risk. In this case series, conditions such as renal impairment and malnutrition were present in a subset of patients, as reported. The retrospective design and limited sample size preclude a formal multivariate analysis to isolate the independent effect of non-adherence while controlling for these variables. Therefore, the association reported between non-adherence and bone marrow suppression, though strong and clinically plausible, may be influenced by these coexisting conditions. Future prospective studies with larger cohorts should systematically collect data on these potential confounders to adjust for their effects and validate the independent role of medication adherence.

## 3 Results

### 3.1 Demographic and baseline characteristics

The clinical characteristics of the patients are summarized in Table 1. A total of 30 patients were enrolled in this study, including 10 males and 20 females. With regard to educational background, 14 patients were illiterate, 8 had primary school education, 5 had middle school education, and 3 had high school education. Blood type distribution was as follows: type A (n = 7), type B (n = 9), type AB (n = 3), type O (n = 3), while blood type was not examined in 8 cases. The mean age was 64.23 ± 12.39 years, body mass index (BMI) was 21.42 ± 2.32 kg/m^2^, and disease duration was 16.16 ± 10.06 years.

**TABLE 1:** Baseline demographic and clinical characteristics of the 30 RA patients with bone marrow suppression.

Item	Patient-related data
Demographic characteristics	
Age (years)	64.23 ± 12.39
Male	58.17 ± 10.54
Female	63.83 ± 10.38
Education level	
Illiterate	14 (46.67%)
Primary school	8 (26.67%)
Middle school	5 (16.67%)
High school	3 (10.00%)
Blood type	
A	7 (31.82%)
B	9 (40.91%)
AB	3 (13.64%)
O	3 (13.64%)
Not tested	8
Baseline data	
BMI(kg/m^2^)	21.42 ± 2.32
Disease duration (years)	16.16 ± 10.06

### 3.2 Manifestations of adverse reactions

Bone marrow suppression occurred in all 30 patients, with 20 cases (66.67%) classified as grade IV. The main accompanying symptoms and complications are summarized in Table 2. Notably, all patients developed oral mucositis/ulceration; 15 cases (50.00%) presented with fever during the agranulocytosis phase; 12 cases (40.00%) had gastrointestinal bleeding; liver injury and renal impairment were each observed in 9 patients (30.00%); skin petechiae or ecchymosis were noted in 5 cases (16.67%); and malnutrition was identified in 17 patients (56.67%).

**TABLE 2:** Concomitant symptoms in 30 RA patients with bone marrow suppression.

Type of adverse reaction	Number of cases [n (%)]
Fever	15 (50.0)
Petechiae/ecchymosis	5 (16.7)
Oral mucositis	30 (100.0)
Gastrointestinal bleeding	12 (40.0)
ALT elevation	9 (30.0)
Renal impairment	9 (30.0)
Malnutrition	17 (56.7)

### 3.3 Clinical outcomes

As shown in Table 3, all 30 RA patients with bone marrow suppression achieved normalization of all three hematopoietic cell lineages following aggressive comprehensive treatment. The time to recovery from bone marrow suppression ranged from 4 to 35 days. All patients showed clinical improvement and were discharged. Additionally, as presented in Table 4, patients with concomitant hepatic or renal dysfunction also demonstrated significant improvement or returned to normal ranges after treatment, except for one case of renal impairment that exhibited transient worsening.

**TABLE 3:** Hematological parameters at admission, time to recovery, and outcomes in 30 RA patients with bone marrow suppression.

Case no.	RBC (×1012/L)	WBC (×109/L)	PLT (×109/L)	HB(g/L)	Recovery time (days)	Outcome
1	2.80	0.21	16	57	18	Improved
2	3.60	2.28	197	98	14	Normalized
3	3.24	0.90	12	71	12	Improved
4	2.64	0.66	66	81	13	Improved
5	2.66	0.70	92	71	12	Improved
6	3.79	0.91	148	97	11	Improved
7	2.68	2.87	12	83	10	Improved
8	1.10	0.84	2	46	10	Improved
9	2.36	0.30	1	69	13	Improved
10	1.95	0.44	3	61	8	Improved
11	2.53	1.59	28	64	6	Improved
12	2.33	0.10	4	59	12	Improved
13	2.58	0.70	14	65	10	Improved
14	2.20	2.13	12	69	7	Improved
15	1.91	0.49	1	57	8	Improved
16	3.21	0.72	17	63	11	Improved
17	3.51	1.99	74	84	9	Improved
18	3.28	0.23	128	90	5	Improved
19	1.56	1.26	18	47	17	Improved
20	2.62	0.76	6	67	35	Improved
21	2.66	2.17	10	79	8	Improved
22	3.49	0.75	227	89	8	Improved
23	2.13	0.93	5	61	4	Improved
24	2.64	1.04	48	71	11	Improved
25	3.01	2.30	15	101	15	Improved
26	3.07	2.11	30	91	11	Improved
27	2.38	0.17	26	65	6	Improved
28	3.32	1.20	14	97	12	Improved
29	2.37	0.05	10	70	7	Improved
30	3.04	1.25	12	99	10	Improved

**TABLE 4:** Changes in liver and kidney function before and after treatment and corresponding outcomes in 30 RA patients with bone marrow suppression.

Case no.	Sex	Scr (μmol/L)		Outcome	Case no.	Sex	ALT (U/L)		Outcome
		Admission	Discharge				Admission	Discharge	
1	M	113	96	Normalized	1	M	86	19	Normalized
8	F	98	82	Normalized	4	F	258	8	Normalized
11	F	101	120	Worsened	5	F	177	105	Improved
14	M	130	104	Normalized	14	M	231	132	Improved
17	M	192	127	Improved	15	M	56	17	Normalized
18	F	106	62	Normalized	18	F	43	20	Normalized
19	F	381	126	Improved	19	F	66	38	Normalized
20	F	144	95	Normalized	20	F	53	12	Normalized
24	F	102	80	Normalized	24	F	235	91	Improved

M, male; F, female.

### 3.4 Analysis of medication adherence

All 30 RA patients received standardized treatment during hospitalization in accordance with the *2018 Chinese Guidelines for the Diagnosis and Treatment of Rheumatoid Arthritis* (Xinping et al., 2021). A comparison between the prescribed dosage of csDMARDs and the actual dosage used is presented in Table 5. Medication non-adherence was identified in 15 patients. Among them, 11 patients exhibited unauthorized increase in dosing frequency, while the remaining 4 patients adjusted both the dosing frequency and the individual dose amount without medical authorization. The other 15 patients adhered strictly to the prescribed dosage.

**TABLE 5:** Comparison of prescribed and actual medication usage of csDMARDs.

Case no.	Prescribed csDMARDs regimen	Actual medication usage	Adherence	Patterns of non-adherence
1	MTX 5 mg once weekly	MTX 5 mg once daily	N	Type A
2	MTX 10 mg once weekly	MTX 10 mg once weekly	Y	-
3	MTX 5 mg once weekly	MTX 10 mg once daily	N	Type A
4	MTX 10 mg once weekly	MTX 10 mg once daily	N	Type A
5	MTX 10 mg once weekly	MTX 10 mg once weekly	Y	-
6	MTX 10 mg once weekly + LEF 20 mg once daily	MTX 10 mg once daily + LEF 20 mg once daily	N	Type B
7	MTX 10 mg once weekly	MTX 10 mg twice daily	N	Type B
8	MTX 7.5 mg once weekly + SASP 1 g twice daily	MTX 7.5 mg once weekly + SASP 1 g twice daily	Y	-
9	GC 15 mg once daily	GC 15 mg once daily	Y	-
10	MTX 7.5 mg once weekly	MTX 7.5 mg once weekly	Y	-
11	MTX 10 mg once weekly	MTX 10 mg once daily	N	Type A
12	MTX 10 mg once weekly	MTX 10 mg twice daily	N	Type A
13	MTX 12.5 mg once weekly	MTX 12.5 mg once weekly	Y	-
14	MTX 10 mg once weekly + HCQ 200 mg twice daily	MTX 10 mg once weekly + HCQ 200 mg twice daily	Y	-
15	MTX 10 mg once weekly	MTX 10 mg twice daily	N	Type A
16	MTX 10 mg once weekly + LEF 20 mg once daily	MTX 10 mg once weekly + LEF 20 mg once daily	Y	-
17	MTX 10 mg once weekly + GC 10 mg once daily	MTX 10 mg once daily + GC 10 mg once daily	N	Type B
18	IGU 25 mg twice daily	IGU 25 mg twice daily	Y	-
19	LEF 20 mg once daily	LEF 20 mg once daily	Y	-
20	MTX 10 mg once weekly	MTX 10 mg once daily	N	Type A
21	MTX 10 mg once weekly + HCQ 200 mg twice daily	MTX 5 mg three times daily + HCQ 200 mg twice daily	N	Type B
22	MTX 2.5 mg once weekly + HCQ 200 mg twice daily	MTX 2.5 mg once weekly + HCQ 200 mg twice daily	Y	-
23	MTX 10 mg once weekly + IGU 25 mg twice daily	MTX 10 mg once weekly + IGU 25 mg twice daily	Y	-
24	MTX 10 mg once weekly	MTX 10 mg once weekly	Y	-
25	MTX 5 mg twice daily	MTX 5 mg twice daily	Y	-
26	MTX 2.5 mg once daily	MTX 2.5 mg once daily	Y	-
27	MTX 10 mg once weekly	MTX 20 mg once daily	N	Type A
28	MTX 10 mg once weekly	MTX 10 mg once daily	N	Type A
29	MTX 5 mg once daily + LEF 10 mg once daily	MTX 5 mg twice daily + LEF 10 mg once daily	N	Type A
30	MTX 10 mg once daily + LEF 20 mg once daily	MTX 10 mg twice daily + LEF 20 mg once daily	N	Type A

MTX, methotrexate; GC, glucocorticoid; SASP, sulfasalazine; LEF, leflunomide; IGU, iguratimod; HCQ, hydroxychlorquine; Type A: Frequency increase only; Type B: combined frequency increase and dosage adjustment.

### 3.5 Analysis of factors associated with medication non-adherence

A univariate analysis compared baseline characteristics between adherent and non-adherent patients (Table 6). While mean age did not differ significantly between groups (61.47 ± 14.99 vs. 67.00 ± 8.77 years, P = 0.205), significant disparities were found in other variables. A substantially higher proportion of non-adherent patients had a disease duration ≥10 years (66.7% vs. 20.0%, *P* = 0.010) and a low educational level (93.3% vs. 53.3%,*P* = 0.013). These results indicate that longer disease duration and lower education are associated with medication non-adherence in this cohort.

**TABLE 6:** Univariate analysis of baseline characteristics by medication adherence status.

Variable	Adherent group (n = 15)	Non-adherent group (n = 15)	Statistical test	Test statistic	p
Age (years), mean ± SD	61.47 ± 14.99	67.00 ± 8.77	Independent samples t-test	t = 0.205	0.205
Disease duration ≥10 years, n (%)	3 (20.00)	10 (66.67)	Fisher’s exact test	χ2 = 0.025	0.01
Education level (low), n (%)	8 (53.33)	14 (93.33)	Fisher’s exact test	χ2 = 0.035	0.013

## 4 Discussion

csDMARDs represent the cornerstone of first-line pharmacological management for RA. Their well-established efficacy, coupled with a favorable safety profile and extensive long-term clinical experience, solidifies their role as the initial treatment of choice for the majority of RA patients without contraindications (Combe et al., 2017; Visser and van der Heijde, 2009; Zhao et al., 2022). Among these MTX is widely regarded as the “anchor drug” in RA therapy (Perrotta et al., 2024). As a folate analog, methotrexate exerts its anti-inflammatory effect primarily by inhibiting the folate metabolic pathway and disrupting DNA synthesis (Friedman and Cronstein, 2019; Zhao et al., 2022). After cellular uptake mediated by the reduced folate carrier 1 (RFC-1), MTX potently inhibits immune-inflammatory responses and suppresses synovial cell proliferation (Cronstein and Aune, 2020), underlying its therapeutic effect in rheumatoid arthritis. However, previous studies have indicated that although MTX is often considered one of the best-tolerated disease-modifying antirheumatic drugs, its narrow therapeutic window nevertheless warrants careful attention. Furthermore, in the context of long-term use, the absence of standardized monitoring protocols and management guidelines can potentially result in severe, and even life-threatening, adverse reactions among RA patients due to inappropriate dosing or individual variations in drug metabolism (Liu et al., 2025; Xu et al., 2022).

The present study revealed that the most severe adverse reaction associated with MTX was bone marrow suppression, which progressed to pancytopenia. All thirty patients developed bone marrow suppression, with twenty cases classified as grade IV (66.7%). All patients also exhibited varying degrees of hepatic dysfunction. Additionally, fifteen patients (50.0%) developed fever during the period of agranulocytosis. Statistical analysis indicated that MTX was the primary causative factor of bone marrow suppression, accounting for 90.0% (27/30) of cases in this cohort. Therefore, strict adherence to clinical protocols regarding MTX indications, dosage, and frequency of administration is imperative. Efforts should also be strengthened to improve patient education on standardized medication use and to reinforce regular monitoring for adverse drug reactions. Previous studies have emphasized the crucial role of active intervention, including intravenous calcium folinate rescue therapy, in the event of MTX-associated adverse reactions. (Howard et al., 2016). However, the present study found that this regimen was not associated with a significant improvement in the clinical recovery process or hematological recovery in some cases. In light of this evidence, intravenous calcium folinate was not routinely administered as a systemic rescue therapy in this study, but was reserved as an individualized oral rinse for patients with specific indications. The suboptimal efficacy of systemic rescue therapy can be influenced by several factors. In particular, the timing of administration is a critical determinant of treatment success. Both premature and delayed delivery outside the optimal therapeutic window can markedly reduce the efficacy of toxicity reversal (Hui et al., 2010); Excessively high doses of calcium folinate may result in “over-rescue”, which not only fails to enhance normal tissue protection but could also antagonize the therapeutic effects of MTX and potentially increase the risk of disease recurrence (Jiang et al., 2022); Another crucial factor is drug stability; calcium folinate is highly susceptible to chemical degradation under inappropriate pH or oxidative conditions, leading to loss of bioactivity (Yibao et al., 2024); Furthermore, patient-specific factors such as impaired renal or hepatic function can significantly alter the pharmacokinetics and clearance of MTX, thereby compromising the efficacy of calcium folinate rescue.

Additionally, other csDMARDs used in combination with MTX, such as LEF, SSZ, HCQ, and GC, may also contribute synergistically to bone marrow suppression. As an inhibitor of dihydroorotate dehydrogenase (DHODH), LEF suppresses the pyrimidine synthesis pathway (Alamri et al., 2021). Its effect superimposes with the folate-antagonizing action of MTX, collectively impairing DNA synthesis in proliferating hematopoietic stem cells and significantly increasing the risk of pancytopenia. The sulfapyridine component of SASP has been associated with dose-related bone marrow suppression, potentially through the induction of apoptosis in hematopoietic progenitor cells (Ye et al., 2024); Although HCQ-induced bone marrow suppression is relatively rare, it may cause unpredictable cytopenia in certain individuals via inhibition of autophagy and lysosomal function (Xiaolin et al., 2025); Long-term or high-dose GC use not only masks signs of infection and delays treatment, but also promotes lymphocytopenia through pro-apoptotic effects and increases the risk of opportunistic infections (Chastain et al., 2024), thereby indirectly exacerbating marrow suppression. Multiple studies indicate that the concomitant use of these drugs with MTX results in myelotoxicity that is not purely additive, but rather exhibits synergistic inhibitory effects through pharmacodynamic and pharmacokinetic interactions (Yang and Mei, 2021). This risk is especially elevated in patients with hepatic or renal impairment or advanced age, necessitating increased clinical vigilance and routine blood count monitoring during combination treatment. However, owing to the limited sample size receiving combination regimens in the present study, further large-scale investigations are needed to clarify their independent effects.

This study highlighted medication non-adherence as a major modifiable risk factor for adverse drug reactions in RA patients with bone marrow suppression, a finding consistent with previous reports (Peter et al., 2021). This study further clarified, through univariate analysis, the patient characteristics closely associated with medication non-adherence. The analysis indicated that, among RA patients who developed bone marrow suppression, a disease duration of ≥10 years and a low educational level were two key factors distinguishing non-adherent from adherent patients. The proportion of patients with a disease duration exceeding 10 years was as high as 66.7% in the non-adherent group, significantly higher than the 20.0% in the adherent group. Concurrently, a low educational level was also significantly more prevalent in the non-adherent group (93.3% vs. 53.3%). This finding holds clear clinical implications: patients with long-standing disease may be more likely to self-adjust their medication due to overconfidence in their own judgment arising from prolonged illness or anxiety regarding disease control; whereas insufficient educational attainment directly limits patients’ ability to comprehend and execute complex dosing regimens, such as once-weekly administration. These two subgroups should therefore be considered priority targets for medication safety education. Although age did not show a statistically significant difference in the univariate analysis, the observed trend toward older age in the non-adherent group suggests that potential influences on adherence—such as polypharmacy and cognitive function—still warrant comprehensive consideration when assessing elderly patients.

This direction of effect suggests that advanced age may influence medication-taking behavior through cognitive decline and the complexity of polypharmacy (Cho et al., 2018; Shaozhen et al., 2025). These non-adherent medication practices ultimately contribute to adverse drug events by altering drug pharmacokinetics, exacerbating drug-drug interactions, and leading to treatment interruptions—all of which can result in drug concentrations falling outside the therapeutic window (Jiang et al., 2025; Sathua and Mohapatra, 2025). Consequently, this study identifies non-adherence—particularly self-escalation of dosage—as a critical yet modifiable risk factor in clinical practice. These findings underscore the importance of reinforcing individualized patient education, with specific emphasis on MTX dosing protocols and its potential severe adverse effects. Notably, the observed frequency of type B blood in this clinical cohort—nine cases among the twenty-three tested patients, accounting for thirty-nine point one percent—appears higher than the twenty-two point zero six percent reported in a prior demographic study of the Shui population in Sandu, Guizhou (Xiao-yan et al., 2004). This discrepancy may highlight the distinction between a general population distribution and a specific clinical cohort selected for a severe adverse reaction. This further suggests that our finding is more likely to reflect the characteristics of a high-risk patient group rather than those of the regional background population, thereby indicating that blood type *per se* is not a dominant risk factor in this context. This study also performed a preliminary analysis of patient geographical and blood group distributions. Regarding blood group distribution, type B was the most frequently observed among tested patients (9 out of 23, 39.1%). However, this finding should be interpreted with caution. The sample size is limited, data were missing for nearly one-quarter of the cohort, and this single-center, descriptive analysis lacks a comparator group. Therefore, no reliable association between blood type and the risk of bone marrow suppression can be inferred from the present data. These preliminary findings require further validation in larger sample sizes. This study enrolled a cohort of RA patients with bone marrow suppression who were admitted to the Department of Rheumatology and Immunology at the Affiliated Hospital of Zunyi Medical University. Although csDMARD-associated bone marrow suppression events were still observed, the overall patient prognosis was favorable, resulting in no mortality—an improvement compared to historical institutional data (Lu et al., 2021).

A high rate of malnutrition (56.67%) was observed in this study, which may be associated with disease activity and medication side effects. The malnourished status may reduce bone marrow reserve and thereby increase the risk of severe bone marrow suppression. This finding highlights the necessity of incorporating nutritional assessment and support into the comprehensive management of RA patients, which itself may represent a modifiable factor for improving prognosis. At the infrastructural level, the rigorous application of enhanced infection control measures—such as mask-wearing, hand hygiene, and environmental management—enabled effective containment of infections even in the absence of laminar airflow facilities, achieving a level of infection prevention comparable to that provided by such specialized environments.

## 5 Conclusion

This study highlights the need for vigilance regarding bone marrow suppression induced by csDMARDs, particularly methotrexate. Concomitant use with other agents and patient non-adherence significantly increase the risk. Through systematic clinical optimization, all patients achieved recovery. However, as a single-center retrospective study with a limited sample size, the findings should be interpreted with caution. The results provide a clinical reference for the safe use of csDMARDs in patients with rheumatoid arthritis.

## Data Availability

The raw data supporting the conclusions of this article will be made available by the authors, without undue reservation.
