# Correction: Clinical characteristics and risk factors of severe myelosuppression in rheumatoid arthritis patients with csDMARD non-adherence: a case series

**DOI:** 10.3389/fphar.2026.1850736

**Published:** 2026-06-03

**Authors:** Xiaoli Pan, Jingqiao Tian, Juan Chen, Yulei Ao, Mei Tian, Anmao Li

**Affiliations:** Department of Rheumatology and Immunology, The Affiliated Hospital of Zunyi Medical University, Zunyi, China

**Keywords:** adverse drug reaction, bone marrow suppression, csDMARDs, medication adherence, methotrexate, rheumatoid arthritis

The article type was erroneously given as Review. The correct article type is Original Research.

The Ethics statement has been added to the original article as “This study was approved by the Ethics Committee of the Affiliated Hospital of Zunyi Medical University”.

Additionally, the Data availability statement has been added to the original article as “The raw data supporting the conclusions of this article will be made available by the authors, without undue reservation”.

The original article has been updated.

